# Homes for Mothers

**Published:** 1920-04-24

**Authors:** 


THE PUBLIC WELL-BEING—(*continued*)

### Homes for Mothers.

A municipal home for mothers will shortly be opened in Birmingham. The work of the health officials shows that there is always a considerable number of mothers who, owing to poor physique, a bad confinement, or adverse domestic circumstances, cannot take up again without grave danger to their health their household duties, and whose circumstances do not permit the rest which would enable them to recuperate. There is danger to themselves from their low state of health, and it reacts also on the welfare of the baby. The municipality has acquired an estate which will give an opportunity of developing the work of meeting these needs in more favourable conditions than hitherto. The estate is pleasantly situated. Twenty-four to thirty mothers will be the guests—paying guests—of the municipality at one time. It is found that in the majority of cases a fortnight's change is sufficient to fit the mother to resume her domestic duties. The guests will be asked to contribute a portion of the cost of their maintenance according to their means, but the scale will be adjusted so that no case which can benefit from a stay at the home will be deterred from going there by the cost. Everything possible will be done to enable mothers to escape from those permanent disabilities which so often follow too early resumption of domestic duties after confinement, and to go back to their homes strong and well, with healthy babies.

The foregoing plans relate to married mothers. An interesting example of what is being done in another centre for unmarried mothers is to be found in Manchester, at Ennismore, Pendleton. In an appeal for funds for this establishment, it is explained that it now contains twelve mothers and twelve babies. The mothers do the work of the home themselves, under the supervision of the matron and a trained nurse. Two of the mothers work in the nursery, one in the laundry, one in the kitchen, and one serves as housemaid. They receive wages for the

THE PUBLIC WELL-BEING—(*continued*).

work they do, and pay out of their wages a sum towards the maintenance of their babies. The other seven girls work at various occupations outside the home. Two are dressmakers, one a bookbinder, two are in domestic service. The intention of those concerned in the management of the home is to keep the girls until their babies are two years old, and then to assist them in finding work which will not necessitate the separation of mother and child. During their stay at the home the girls are taught how to look after their children and feed them. The girls who work outside the home, like those who do the work within it, contribute from their wages to their children's upkeep.

A home for unmarried mothers and their babies is shortly to be opened at Blackburn.

